# Human Albumin and N-Acetylcysteine for treatment of Fat Embolism: A Case Report

**DOI:** 10.31729/jnma.4478

**Published:** 2019-08-31

**Authors:** Niraj Kumar Keyal, Manish Nakarmi, Amid Bhujel, Sanjeev Kumar Yadav

**Affiliations:** 1 Department of Critical Care Medicine, B & C Medical College and Teaching Hospital, Birtamode, Nepal

**Keywords:** *fat embolism*, *human albumin*, *N-Acetylcysteine*

## Abstract

Fat embolism is a life-threatening condition that mostly occurs after long bones and pelvis fractures and treatment is controversial with many available drugs. We hereby present a case of 53 years male who developed shortness of breath, tachycardia, fever, anemia, distended jugular vein, thrombocytopenia, hypoalbuminemia and was diagnosed to have fat embolism after fracture of femur, tibia, fibula and pubic rami following road traffic accident. Patient was treated with 20 percent human albumin, N-acetylcysteine, other supportive treatment and discharged after fourteen days. From this we want to emphasize role of human albumin and N-acetylcysteine in treatment of fat.

## INTRODUCTION

Fat embolism is presence of fat globules in microcirculation and lung parenchyma. It commonly occurs following trauma to long bones and pelvis in 95% and it is non traumatic in 5%.^1^ The incidence ranges from 0.25% to 33%. It usually occurs within 12 to 72 hours but it can occur as early as 6 hours to 10 days.^2^ Fat embolism has mortality of 10-15% due to its lack of specific symptom and treatment.^3^ Gurd's, Lindeque's, Schonfeld criteria are used to diagnose fat embolism. This case report shows successful treatment with controversial drugs like Albumin and N-acetylcysteine.

## CASE REPORT

**Figure 1:**
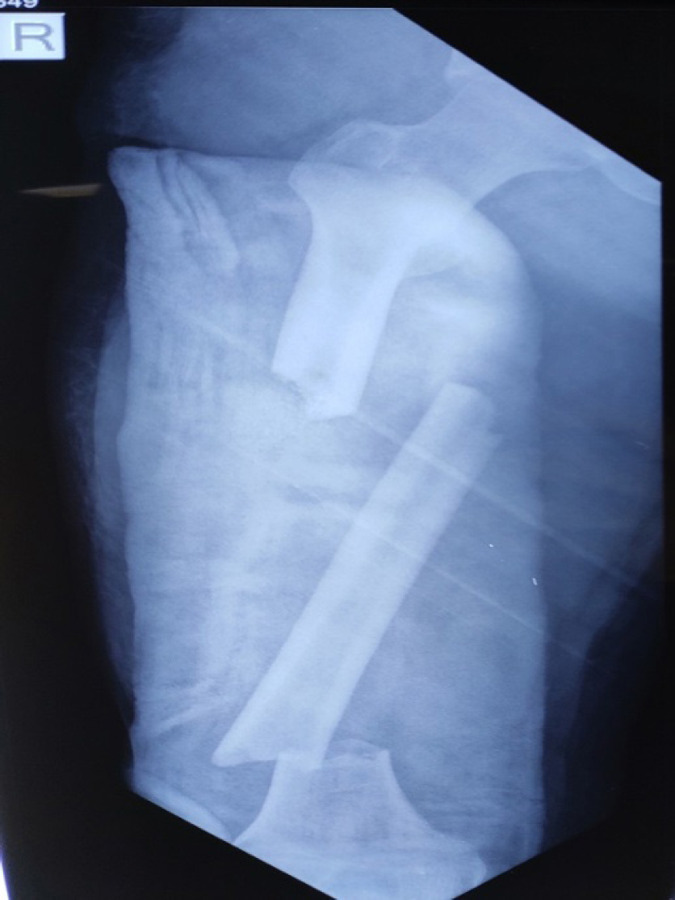
X-ray showing right femur fracture.

**Figure 2.**
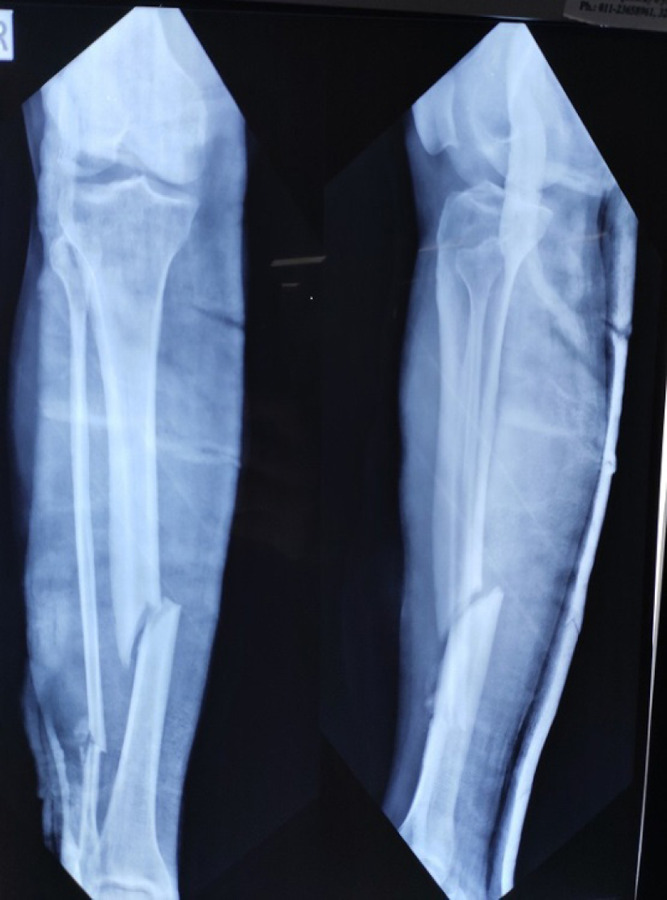
X-ray showing right tibia and fibula fracture.

A 53 years male, without any significant past history presented to an emergency department after sustaining injury at lower limb, abdomen and chest. At presentation, his Glasgow Coma Scale (GCS) was 14/15 with pulse rate of 140 beats/per min, blood pressure of 70/40 mmHg, respiratory rate of 21breaths/min, oxygen saturation of 94% on 10 liter oxygen. Primary and secondary survey was done which showed fracture of right sided femur, tibia and fibula (Figure 1, 2). Chest compression was negative. Immediately, patient was resuscitated with blood products and other supportive management. Examination of chest showed decreased air entry on left side. Per abdomen and central nervous system examination were normal.

**Figure 3.**
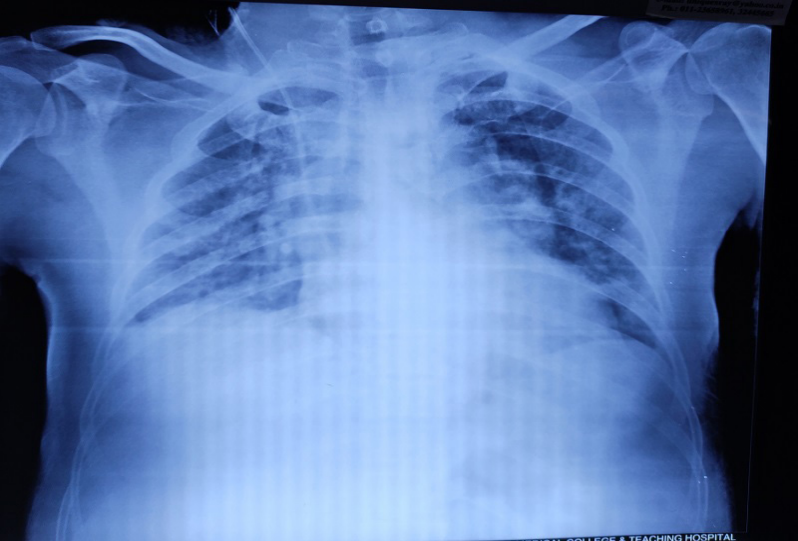
Chest X-ray anteroposterior view showing bilateral infiltrates.

His investigation profiles were Total leucocyte count- 15000/mm^3^, Platelets- 100000/mm^3^, Hemoglobin (Hb)- 7 gm/dl, Hematocrit- 21%, Urea- 90 mg/dl, Creatinine- 1.8 mg/dl, Sodium and Potassium were normal. Total bilirubin was 2.2 mg/dl in which direct bilirubin was 1.1 mg/dl with total protein of 5.9 mg/dl in which albumin was 5.1 mg/dl, Alanine aminotransferase (ALT) was 201 U/L, Aspartate aminotransferase (AST) was 281 U/L. Patient was resuscitated with blood and other supportive treatment. Intra-medullary nailing and fixation of femur and tibia was done on second day of trauma. Renal function and liver function improved after resuscitation. On 3^rd^ day, patient suddenly developed shortness of breath, fever 102^0^ F and tachycardia, tachypnoea, saturation was 84% on 15 liter of oxygen and jugular venous pressure was 12 cm H_2_0. Blood investigation showed hemoglobin 7 gm/ dl, platelets-100000/mm^3^, Prothrombin Time- 21 sec, International Normalized Ratio- 2.1, calcium- 7 mg/ dl, albumin- 3 mg/dl and chest x-ray showed bilateral infiltrates (Figure 3). Arterial blood gas analysis showed mild hypoxemia with hypocarbia, respiratory alkalosis and lactic acidosis. Transthoracic echocardiography showed moderate tricuspid regurgitation and peak gradient was 15 cm/s. Patient was diagnosed as fat embolism based on Schonfeld criteria. Petechial rash was also seen but altered sensorium was not seen in our patient. Bronchoalveolar lavage and demonstration of fat globules in urine and macrophages was not done as it was not available at our center.

Patient was started on Meropenem, Doxycycline, 20 percent human albumin 100 ml, nine gram (150mg/kg) of N-acetylcysteine over 4 hours, furosemide, calcium and other supportive treatment. Patient was followed and gradually albumin rose to 4 mg/dl on 5^th^ day of treatment and hemoglobin, platelet, dyspnoea gradually improved on 6^th^ day. Low molecular weight heparin was started on 6^th^ day for thromboprophylaxis. Patient was transferred out of intensive care unit (ICU) on 7^th^day and was discharged on 14^th^ day.

## DISCUSSION

Fat embolism is misdiagnosed due to co-existence of other injuries, shock lung, lung contusion and non specific symptom. There is a lack of specific clinical symptoms and laboratory parameters for diagnosis of fat embolism therefore different criteria are used to diagnose fat embolism but none of them are specific. Treatment is usually supportive due to unavailability of specific treatment and treatment is controversial with methylprednisone, heparin, aspirin, n-acetylcysteine, albumin and inferior vena caval filter.^4^

Fat embolism can be prevented by early fixation of fractures, albumin infusion in operation theatre and venting of medullary cavity during surgery. Early fixation and albumin infusion was not done in our patient. Preventive measures should be taken in patients who have independent risk factor for developing fat embolism. In our patients preventive measures were not taken.

Studies have shown that hypoalbuminemia is usually present in fat embolism and albumin can be used for volume resuscitation and as a prophylaxis as albumin can bind free fatty acids and decrease the incidence of fat embolism and lung injury^4^ but role in treatment in fat embolism is shown in rats^5^ but there is no study in human. Large scale studies are required to identify the role of albumin in treatment. We used albumin in our patient that gave better result.

Studies in rats have shown N-acetylcysteine causes decrease in lung injury.^6^ There were no studies in humans to show its benefit but was used in our patients as N-acetylcysyeine has no major side effects. Large scale study is required to know effects in human.

To conclude, preventive measures should be taken in patient with risk of fat embolism and large scale studies are needed to evaluate effects of human albumin and N-acetylcysteine in fat embolism.

## Consent:

**JNMA Case Report Consent Form**was signed by the patient and the original is attached with the patient's chart.

## Conflict of Interest:

**None.**
